# Restoring High Vaccine Coverage in Brazil: Successes and Challenges

**DOI:** 10.1590/0037-8682-0614-2023

**Published:** 2024-02-12

**Authors:** Eder Gatti Fernandes, Guilherme Loureiro Werneck, Ana Estela Haddad, Ethel Leonor Noia Maciel, Nísia Verônica Trindade Lima

**Affiliations:** 1 Ministério da Saúde, Secretaria de Vigilância em Saúde e Ambiente, Departamento do Programa Nacional de Imunizações, Brasília, DF, Brasil.; 2 Ministério da Saúde, Secretaria de Vigilância em Saúde e Ambiente, Departamento de Ações Estratégicas de Epidemiologia e Vigilância em Saúde e Ambiente, Brasília, DF, Brasil.; 3 Ministério da Saúde, Secretaria de Informação e Saúde Digital, Brasília, DF, Brasil.; 4 Ministério da Saúde, Secretaria de Vigilância em Saúde e Ambiente, Gabinete da Secretária de Vigilância em Saúde e Ambiente, Brasília, DF, Brasil.; 5 Ministério da Saúde, Gabinete da Ministra da Saúde, Esplanada dos Ministérios Bloco “G” Térreo, Brasília, DF, Brasil.

Facing the decline in vaccine coverage observed since 2016, the government under President Lula committed to reversing this scenario and has prioritized the resurgence of high vaccine coverage in Brazil. To achieve this, the Federal Government launched the National Vaccination Movement (*Movimento Nacional Pela Vacinação*) in early 2023 and directed all technical and communication actions of the Ministry of Health to promote population vaccination with the slogan 'vaccine is life, vaccine is for everyone.' Alongside the National Vaccination Movement, the government: (1) launched the 'Health with Science' platform to monitor and combat vaccine misinformation, (2) promoted microplanning, providing tools for decision-making at the territorial level and allocating 151 million reais (approximately 30 million dollars) to the states and municipalities to implement innovative vaccination strategies, and (3) standardized the rules of information systems for registration of vaccine doses and directed all data to the National Health Data Network (*Rede Nacional de Dados em Saúde* - RNDS).

Reversing this declining vaccination coverage trend in Brazil is a challenge; however, it is possible to observe significant results from government actions within just one year. Our initiatives were accompanied by efforts from the Legislative branch by the creation of the National Front for Vaccination (*Frente Nacional pela Vacinação*), the National Council of Prosecution Services (CNMP) with the National Pact for Vaccine Awareness (*Pacto Nacional pela Consciência Vacinal*), the National Council of Municipal Health Secretariats (Conasems), the National Council of Health Secretaries (Conass), the Oswaldo Cruz Foundation (Fiocruz), states, municipalities, scientific associations, and organized civil society.

Of the eight recommended vaccines by the age of one year, seven vaccines showed an increase in vaccination coverage in 2023 throughout Brazil when compared to the coverage recorded in 2022: Hepatitis A, pneumococcal, meningococcal, poliomyelitis, diphtheria-tetanus-pertussis (DTP), and the first, and second doses of the measles, mumps, and rubella (MMR) vaccine. Nationally, the vaccination coverage has increased from 4.0% (2^nd^ dose of MMR vaccine from 57.6% in 2022 to 61.6% in 2023) to 7.8 percentage points (pneumococcus, from 71.5% in 2022 to 78.0% in 2023).

When evaluating the vaccination coverage across states, all states showed improvements in the DTP vaccine coverage. Twenty-six states showed increased coverage for polio and the first dose of the MMR vaccine. Twenty-four states expanded coverage for hepatitis A, meningococcal, and first dose of MMR vaccine, whereas 23 states showed enhanced coverage for the pneumococcal vaccine. 

In this scenario of reversing the declining vaccination coverage in Brazil, the state of Piauí stood out by increasing coverage for the first dose of the MMR vaccine from 73.1% to 97.8%, for polio from 75.9% to 89.0%, and for DTP from 73.1% to 92.8%. The state of Espírito Santo has shown an increase in the meningococcal vaccine coverage from 75.9% to 89.9%. In Rondônia, the vaccination coverage for the first dose of the MMR vaccine increased from 89.2% to 99.6%, reaching the recommended target.

The increase in vaccination coverage directly affected the number of municipalities meeting the recommended goals. Among the vaccines recommended for one-year-old children, the DTP vaccine stands out, with an additional 713 municipalities meeting the 95% vaccination coverage goal (from 1,467 in 2022 to 2,180 municipalities in 2023), followed by the polio vaccine, with an increase of 705 municipalities meeting the goal (from 1,463 to 2,168 municipalities), and hepatitis A, with an expansion of 701 municipalities (from 1,745 to 2,446 municipalities).

The yellow fever vaccine recommended at nine months of age stood out among those recommended for children under one year. Its coverage has increased from 60.7% in 2022 to 67.3% in 2023; and all states have reported increased vaccination coverage. The Federal District was one of the highlights, progressing from 71.9% in 2022 to 82.9% in 2023. Rio Grande do Norte and Sergipe recorded absolute increases in yellow fever vaccine coverage of >30%, reaching levels close to the target level.

A significant highlight regards the human papillomavirus (HPV) vaccine, which had been experiencing a decline in the number of doses administered since 2014 despite the increase in the population to which the vaccine should be administered^1^ ^,^ ^2^. In 2023, unlike in the previous years, there was a 30% increase in HPV vaccination. In addition to microplanning, vaccination in schools, promoted by several municipalities, was a fundamental strategy for this positive vaccination result.

The recovery of vaccine coverage recommended for children under six months of age represents a significant challenge. More than in other age groups, achieving high vaccine coverage in children under six months depends on the primary care actions for early childhood^3^ ^,^ ^4^. The restoration of vaccination coverage in this age group was related more to primary care coverage than to the intensification of vaccination efforts^4^. When these children are not appropriately vaccinated until they reach one year, the catch-up doses are not included in the vaccination coverage for children <1 year. Therefore, vaccine coverage for recommended vaccines between 2-6 months of age (rotavirus, meningococcal, pentavalent, pneumococcal, and poliomyelitis) remains stable when compared to the coverage in 2022.

Continuous monitoring of vaccine coverage is essential for the success of national immunization programs. Therefore, a comprehensive, flexible, and timely vaccine information system is required. Restructuring of vaccine information systems is urgently required. This was also the subject of action in 2023, with all vaccine data directed towards the RNDS.

Until 2022, the records of routine vaccine doses administered were consolidated from various information systems and presented in the Tabnet panel. Once within the RNDS, all doses were linked to an individual's Brazilian registry number (*Cadastro de Pessoa Física*, CPF), a recommendation made by the World Health Organization, and ultimately adopted by Brazil. This allowed the digital vaccination card to become a reality: the citizens can check their vaccination status online on ConecteSUS (https://conectesus-paciente.saude.gov.br/login) for all vaccines.

All data from the RNDS are available on a panel of administered doses and vaccine coverage, which directly consumes information from the National Health Data Network. Currently, the panel displays coverage for vaccines recommended for children under two years of age. Although the panel does not yet show vaccine coverage for individuals older than two years, it displays the number of doses administered for all vaccines.

To migrate the data to the RNDS, the old Information System of the National Immunization Program (SIPNI web or "Legacy") had its data input stopped, transferring routine dose records made outside primary care to the new SIPNI system, already used for registering COVID-19 vaccine doses and directly integrated into the RNDS. This occurred on June 1, 2023, affecting the recording of BCG and hepatitis B vaccine doses typically administered in maternity wards. The 2.6 million doses administered from January to May 2023 and recorded in the legacy SIPNI still need to be uploaded to the RNDS. Among these, 400,000 were BCG doses and 600,000 were hepatitis B doses. Hence, they were not included in the calculation of vaccination coverage. Consequently, the national vaccination coverage for these two vaccines remains below the target: 61.4% and 55.5% for BCG and hepatitis B, respectively. The incorporation of the doses retained in the “Legacy” SIPNI is likely to increase the vaccination coverage of such vaccines. By the first semester of 2024, all the data will be incorporated into the RNDS.

In migrating data from the Primary Care Information System (*Sistema de Informação da Atenção Básica*, SISAB) to the National Network of Health Data (RNDS), 6 million out of 100 million administered doses were retained. These records did not match the vaccinated individual's CPF (Individual Registry Number) or National Health Card (*Cartão Nacional de Saúde* - CNS). Most retained records were for vaccines recommended in the first six months of life: inactivated polio (707,438 doses), pentavalent (707,720 doses), 10-valent pneumococcal (607,331 doses), rotavirus (527,496 doses), meningococcal C (512,988 doses), BCG (288,065 doses), and hepatitis B vaccine (322,557 doses).

By migrating these records to the RNDS, the vaccination coverage rates better reflect the current situation. For instance, the pneumococcal vaccine coverage in <1-year-olds in 2023 increased from 78.8% to 83.4%. The Ministry of Health team, in collaboration with states and municipalities, is working to rectify these records and submit them to the RNDS so that these doses are included and accounted for in the calculation of vaccination coverage. Addressing inconsistencies in vaccine doses and directly feeding them into the RNDS represents a significant vaccination effort in the country, which is achievable through collaborative work with states and municipalities.

Other factors may have contributed to the calculated vaccination coverage for 2023, yet to reflect the gains achieved or hindering even more significant progress. First, the panel calculates the vaccine coverage with doses administered up to two months ago (today, up to October 2023). Municipalities with their own information systems or those that operate offline may take up to four months to send their data on the doses administered. Second, during the second half of 2023, there was a varicella vaccine shortage due to the sanitary suspension of batches^5^. Finally, there was a temporary shortage of meningococcal C vaccines.

Despite these improvements, coverage targets are yet to be achieved. The maintenance and expansion of the adopted strategies, with actions targeted at the territory, vaccination in schools, and strengthening of primary care will ensure a steady increase in and recovery of vaccination coverage in the short-term.

As outlined above, several challenges remain in advancing the vaccination agendas. However, there are reasons to celebrate the reversal of the declining trend in the coverage of several vaccines in the first year of the government and increase in number of municipalities that have fully achieved the vaccination coverage target. The National Vaccination Movement, with public opinion awareness, the resumption of regionalized communication campaigns, localized micro-planning actions, including various extramural vaccination strategies, such as the participation of 3,992 municipalities that conducted vaccinations in schools, and integration of the information systems, has been decisive in strengthening work in the territories, thus restoring a culture of immunization, a source of pride in the country, and internationally recognized throughout the 50 years of the National Immunization Program.

## Data Availability

Until the vaccination panel is not launched, the data are available on demand to the Departamento do Programa Nacional de Imunizações. Email: pni@saude.gov.br / phone: (61) 3315-3874.
